# Mutations that impair Eyes absent tyrosine phosphatase activity *in vitro* reduce robustness of retinal determination gene network output in *Drosophila*

**DOI:** 10.1371/journal.pone.0187546

**Published:** 2017-11-06

**Authors:** Trevor L. Davis, Charlene S. L. Hoi, Ilaria Rebay

**Affiliations:** 1 Ben May Department for Cancer Research, The University of Chicago, Chicago, Illinois, United States of America; 2 Committee on Development, Regeneration and Stem Cell Biology, The University of Chicago, Chicago, Illinois, United States of America; 3 Committee on Genetics, Genomics and Systems Biology, The University of Chicago, Chicago, Illinois, United States of America; University of Florida, UNITED STATES

## Abstract

A limited collection of signaling networks and transcriptional effectors directs the full spectrum of cellular behaviors that comprise development. One mechanism to diversify regulatory potential is to combine multiple biochemical activities into the same protein. Exemplifying this principle of modularity, Eyes absent (Eya), originally identified as a transcriptional co-activator within the retinal determination gene network (RDGN), also harbors tyrosine and threonine phosphatase activities. Although mounting evidence argues for the importance of Eya’s phosphatase activities to mammalian biology, genetic rescue experiments in *Drosophila* have shown that the tyrosine phosphatase function is dispensable for normal development. In this study, we repeated these rescue experiments in genetically sensitized backgrounds in which the dose of one or more RDGN factor was reduced. Heterozygosity for *sine oculis* or *dachshund*, both core RDGN members, compromised the ability of phosphatase-dead *eya*, but not of the control wild type *eya* transgene, to rescue the retinal defects and reduced viability associated with *eya* loss. We speculate that Eya’s tyrosine phosphatase activity, although non-essential, confers robustness to RDGN output.

## Introduction

Only tens of signaling pathways and roughly one thousand transcription factors operate in metazoan species [1–3]. One means of increasing regulatory potential is to incorporate feedback that dampens or amplifies signaling flux depending on context [4]. Another strategy is to evolve modular proteins with a physically separable set of biochemical capabilities that can be harnessed in different combinations appropriate to specific developmental, cellular or subcellular situations [5].

*Drosophila* Eyes absent (Eya), a transcriptional co-factor and protein phosphatase, provides an opportunity to study how multifunctional proteins integrate and deliver regulatory information. Eya family proteins are highly conserved throughout metazoans [6–11]. In the nucleus, Eya’s C-terminal Eya domain (ED) binds to the homeodomain transcription factor Sine oculis (So), referred to as Six in vertebrates, while its N-terminal transactivation domain (TAD) confers transcriptional activation ability to the complex [12–14]. Along with the other two core members of the retinal determination gene network (RDGN), Eyeless (Ey) and Dachshund (Dac), the *Drosophila* Eya-So transcription factor controls the expression of target genes that direct many steps in eye formation, including establishment of regional identity, proliferation, specification, and differentiation [15–20].

Less understood are Eya’s two protein phosphatase activities: a threonine phosphatase domain sandwiched between two portions of the N-terminal TAD [21,22] and a tyrosine phosphatase whose key catalytic residues are dispersed in linear sequence across the ED [23–25]. Focusing on the tyrosine phosphatase, initial overexpression experiments in *Drosophila* suggested functional relevance [23,24,26], but a subsequent loss-of-function based analysis concluded that Eya’s tyrosine phosphatase activity is not required for normal *Drosophila* development [27]. Specifically, using fully functional genomic BAC rescue transgenes, Jin et al. showed that introducing missense mutations biochemically proven to disrupt tyrosine phosphatase function in vitro and in mammalian cultured cells did not compromise development, fertility, or survival of the fly [27]. In light of the identification of physiologically relevant substrates for mammalian Eya tyrosine phosphatases [28–31], together with the high degree of evolutionary conservation of the residues that form the phosphatase catalytic core, we found the dispensability of Eya’s tyrosine phosphatase activity in the fly puzzling [32].

Here, we tested the alternative model that Eya’s tyrosine phosphatase function confers robustness to RDGN regulatory output by re-examining the genetic rescue capability of the phosphatase-dead *eya* BAC transgene under conditions of genetic stress. Sensitizing the network with *eya*, *so* and/or *dac* heterozygosity, neither of which on its own or in combination caused overt phenotypes, revealed that “phosphatase-dead” *eya* lacked sufficient activity to support normal retinal development and survival. While the most parsimonious interpretation is that Eya’s tyrosine phosphatase activity contributes to overall RDGN output, until physiologically relevant substrates are identified, an equally plausible possibility is that the “phosphatase-dead” mutations disrupt some other function or interaction. We hope our work will motivate further study of Eya’s possible enzymatic activities in *Drosophila*.

## Materials & methods

### *Drosophila* strains and crosses

The *eya*^*+*^*GR*, *eya* ^*D493N*^*GR*, and *eya* ^*E728Q*^*GR* BAC transgenes, each integrated in the AttP2 (68A) landing site, were generous gifts from Graeme Mardon [27]; the fly strains were sent to us in an *eya*^*cli*^ background and were crossed to *Sco/CyO*,*act-GFP; Sb/TM6B*,*Hu*,*Tb* to establish *eya*^*cli*^/*CyO*,*act-GFP; GR/TM6B*,*Hu*,*Tb* stocks from which eya^cli^/*CyO*,*act-GFP; eyaGR* were crossed to: 1) eya^cli^/*CyO*,*dfd-YFP* 2) *eya*^*cli*^,*so*^*3*^*/CyO*,*dfd-YFP* 3) *eya*^*cli*^,*dac*^*3*^*/CyO*,*dfd-YFP* 4) 5) *eya*^*cli*^, *dac*^*3*^,*so*^*3*^*/CyO*,*dfd-YFP* 6) *eya*^*A188*^,*so*^*3*^*/CyO*,*dfd-YFP* 7) *Df(eya)/CyO*,*dfd-YFP*. *CyO*,*dfd-YFP* was a gift from Greg Beitel. *eya*^*A188*^ and *eya*^*G130*^ were isolated by Rebay et al., 2000 [33] and characterized in Bui et al., 2000 [8]. *eya*^*cli*^, *so*^*3*^, *dac*^*3*^, *Df(2L)BSC354 (Df(eya))*, *w*^*1118*^ and various balancers were from the Bloomington Stock Center. Flies were cultured on standard cornmeal, molasses, agar medium at 25°C.

For the genetic rescue experiments, crosses were set with 2–3 adult flies of each sex, allowed to lay eggs for 3–4 days, transferred to a fresh vial for another 3–4 days before discarding the parents. Adult progeny were counted daily until all had eclosed. Survival was measured by comparing the expected Mendelian ratio of rescued straight winged *eya*^*cli*^*;GR/+* animals relative to their curly winged (*eya*^*cli*^*/CyO*,*act-GFP;GR/+*) siblings. Crosses were performed at least five times in parallel.

To assess female fertility, rescued female flies (*eya*^*cli*^*;GR/+*) were collected as virgins, fed yeast for 2d at 25°C, and then crossed in pairs to three *w*^*1118*^ males. Parents were tossed after 9 days and progeny were counted daily until all flies eclosed. Ten crosses were set in parallel, but only those in which both females survived were scored.

### Immunohistochemistry and microscopy

For antibody staining, third instar eye-antennal imaginal discs were dissected in S2 cell medium, fixed for 10 min in 4% paraformaldehyde with 0.1% Triton X-100, washed 3X in PBT (1X PBS, 0.1% Triton), blocked in PNT (1X PBS, 0.1% Triton, 1% normal goat serum), stained with primary antibodies in PNT overnight at 4° C, washed 3X in PBT, and stained with secondary antibodies in PNT for 2 h at room temperature or overnight at 4° C. Primary antibodies were rat α-ELAV (1:50, Developmental Studies Hybridoma Bank [DSHB], 7E8A10) [34] and mouse α-Eya (1:10), DSHB, 10H6 [6]. Secondary antibodies were donkey α-rat-Cy3 (1:2000) and donkey α-mouse-Cy3 (1:2000) from Jackson Immunoresearch. We used DAPI (1:2000, Invitrogen) to detect DNA. Imaging was performed with a Zeiss LSM 510 confocal microscope, using 0.5 to 1.0 μm steps and projecting maximally through the desired tissue.

To image adult eyes, 3–5 day old adult flies were decapitated and photographed with a Canon EOS Rebel camera fitted to a Leica dissecting microscope. Individual slices were merged using iSolution-Lite software (IMT-Digital).

### Adult retina embedding and sectioning

Adult heads were decapitated, halved, fixed for 30 min in 2.5% glutaraldehyde + 1% O_S_O_4_ in 0.1 M sodium phosphate buffer pH 7.2 for 30 min on ice, and incubated in 2% O_S_O_4_ in 0.1 M sodium phosphate buffer pH 7.2 for 2h on ice. Retinas were then dehydrated with successive 10 min incubations on ice in 30%, 50%, 70%, 90%, and twice 100% EtOH. The tissue was incubated 3x 10 min on ice in propylene oxide, placed in 50% propylene oxide/50% soft Durcupan resin (Sigma) for 12h at room temperature, and then in 100% resin for 4h at room temperature. Retinas were then transferred to BEEM flat embedding molds (Ted Pella) and baked at 70°C for 12h, cut into 2 μm sections with a microtome, and mounted in DPX (Sigma). Samples were imaged at 100x under phase contrast.

## Results and discussion

### Lowering *so* and *dac* levels reveals a requirement for Eya’s tyrosine phosphatase activity during retinal development

Under physiologically wild type conditions, Eya’s tyrosine phosphatase activity is dispensable for development and survival [27]. In contrast, overexpression-based genetic rescue and ectopic eye induction assays have found that the same phosphatase-dead mutations diminish or ablate function, suggesting the phosphatase can function in *Drosophila* cells [23,24,26]. This discrepancy led us to hypothesize that there might be sufficient regulatory redundancy within the RDGN to compensate for loss of phosphatase activity under physiological conditions but not under overexpression conditions. If so, then lowering the dosage of other RDGN factors, which on its own does not compromise normal development or survival, might sensitize the system to reveal the essential contributions of the phosphatase. We selected So and Dac to test this idea because of their intimate genetic and physical relationship with Eya in the RDGN [6,35–38], The three genomic rescue (GR) transgenes used in our experiments, *eya*^*+*^*GR*, *eya*^*E728Q*^*GR* and *eya*^*D493N*^*GR*, were generous gifts from Graeme Mardon and colleagues [27].

**Fig 1:**
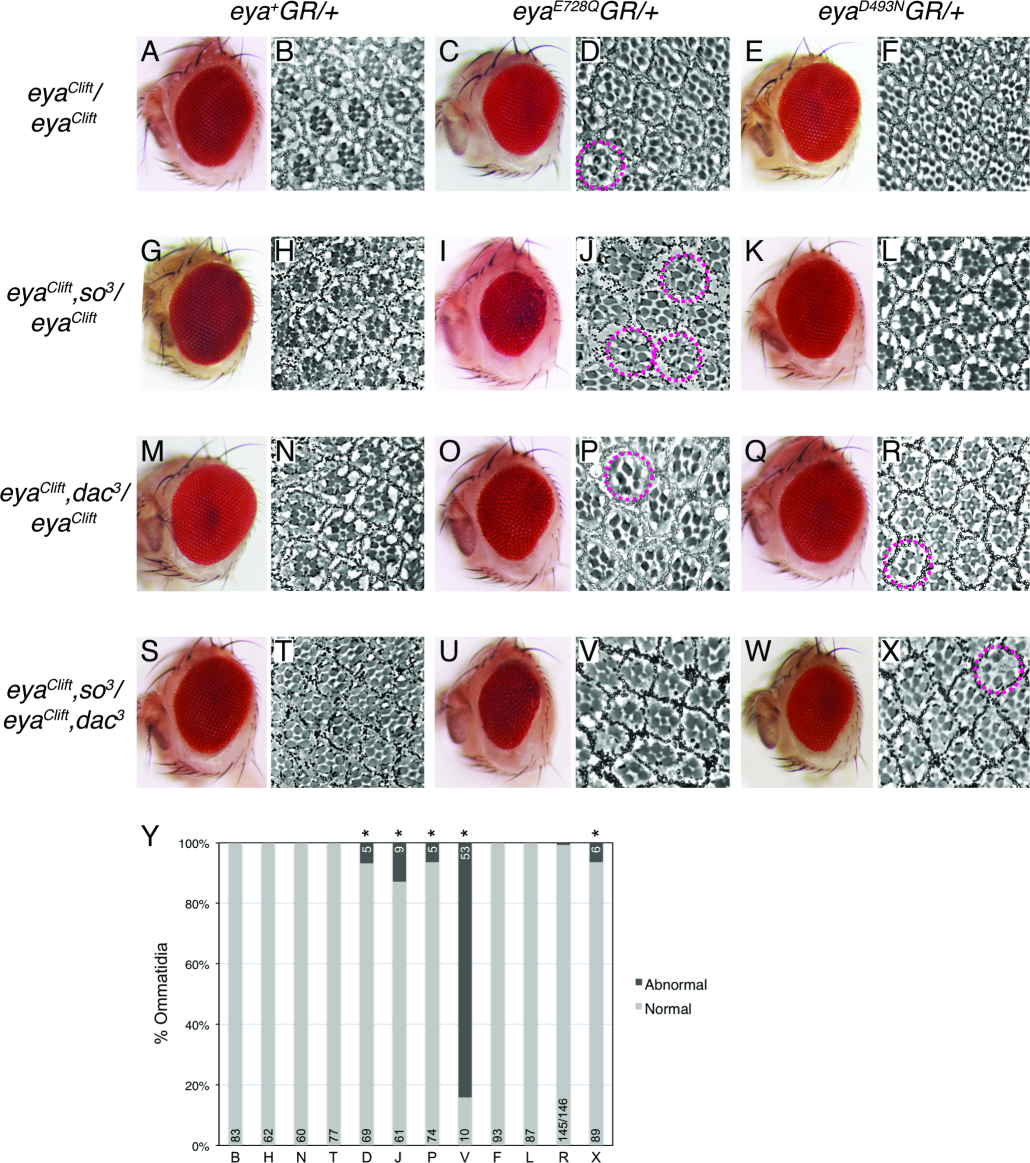
*so* and *dac* heterozygosity reveals that Eya’s tyrosine phosphatase functions during retinal development. Genotypes on the left refer to the second chromosome, while those on the top refer to the third chromosome. (A,C,E,G,I,K,M,O,Q,S,U,W) Low resolution images of representative adult eyes from three-day-old flies of the indicated genotypes; for each genotype, qualitative examination of a minimum of 400 adults (from multiple independent crosses) under the dissecting microscope revealed all phenotypes were fully penetrant. (B,D,F,H,J,L,N,P,R,T,V,X) Sections from representative adult eyes of the indicated genotypes. Pink dashed circles mark individual ommatidia lacking the full complement of rhabdomeres, except in V where all ommatidia in the field have missing rhabdomeres. All images are oriented anterior to the left. (Y) A minimum of 60 ommatidia were scored from sections of the same eyes shown in (B,D,F,H,J,L,N,P,R,T,V,X); ommatidia were scored as normal if they had the wild type complement of rhabdomeres and abnormal if they did not. Statistical significance was assessed with a Fisher’s exact test; * indicates P < 0.05.

We first assayed Eya’s tyrosine phosphatase contribution to retinal development by rescuing *eya* loss with GR transgenes in genetically sensitized backgrounds and analyzing adult eye morphology. In the control experiment, heterozygosity for *so* or *dac* or for both *so* and *dac* did not alter the ability of one copy of the *eya*^*+*^*GR* wild type rescue transgene to support normal eye morphology in an *eya*^*cli*^ null background (Fig 1, first two columns). Flies rescued with two copies of any of the three GR transgenes were indistinguishable from wild type (Fig 1A) in these heterozygous backgrounds. In contrast, in all three sensitized backgrounds, the external morphology of eyes from flies rescued with one copy of the phosphatase-dead *eya*^*E728Q*^*GR* or *eya*^*D493N*^*GR* transgenes was no longer fully wild type (Fig 1, last four columns). Across the board, qualitative assessment of these fully penetrant rough eye phenotypes indicated that *eya*^*E728Q*^*GR* rescued *eya* loss less efficiently than *eya*^*D493N*^*GR*. Phenotypically, in the eyes of the former, the regularity of the ommatidial lattice was noticeably disrupted when viewed under a dissecting microscope, and upon sectioning, some photoreceptor loss was apparent. In contrast, eyes rescued by *eya*^*D493N*^*GR* exhibited mild disruption of the ommatidial lattice, particularly near the posterior margin and less frequent photoreceptor loss. Based on qualitative assessment of the adult rough eye phenotypes, for both *eya*^*E728Q*^*GR* and *eya*^*D493N*^*GR*, reducing the dose of *so* sensitized the system more effectively than reducing the dose of *dac* (Fig 1, compare middle two rows), while the *so*, *dac* double heterozygote heightened sensitivity to loss of phosphatase activity (Fig 1, bottom row). To rule out the possibility that the phenotypes reflected interactions with secondary mutations on the *eya*^*cli*^ chromosome, we repeated all of the crosses shown in Fig 1 in different transheterozygous backgrounds, including the same *eya*^*cli*^*/Df(eya)* used by Jin et al., 2013, *eya*^*cli*^*/eya*^*G130*^ and *eya*^*cli*^*/eya*^*A188*^, and observed identical results (Fig 1). We propose that when Eya, So and Dac are limiting, Eya’s tyrosine phosphatase activity may be required to achieve sufficient RDGN output to support normal retinal development.

**Fig 2:**
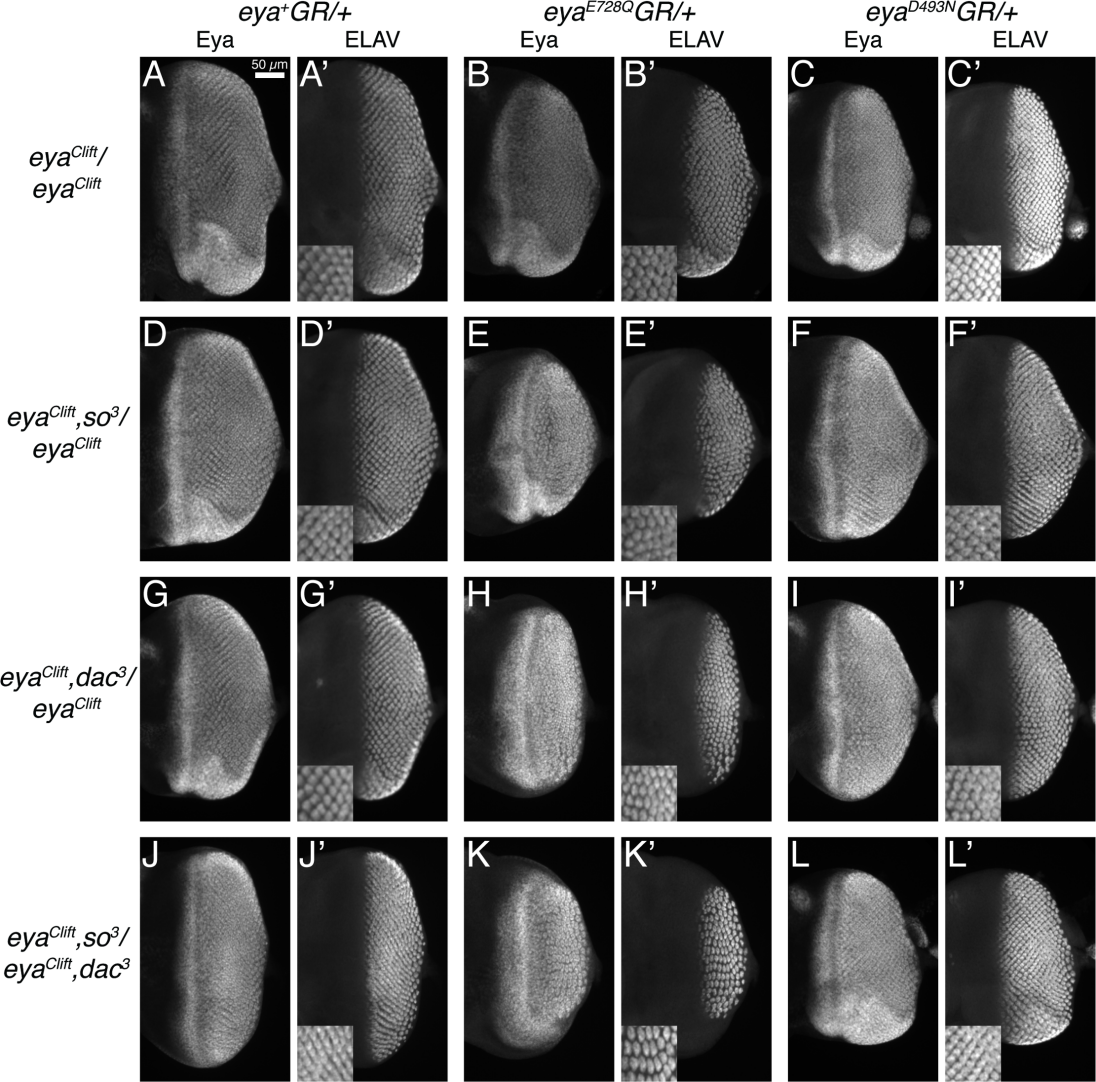
Retinal phenotypes in animals lacking Eya’s tyrosine phosphatase activity and heterozygous for *so* or *dac* manifest by the third larval instar. All images are maximum projections through representative late third instar eye-antennal imaginal discs. Anterior is to the left and dorsal is up. Only the eye disc is shown. ELAV marks cells undergoing neuronal differentiation, and insets show zoomed views of the ELAV channel. Genotypes on the left refer to the second chromosome, while those on the top refer to the third chromosome. The markers are listed above each column of images. For each genotype, qualitative examination of a minimum of 15 discs revealed fully penetrant phenotypes.

To pinpoint more precisely when Eya’s tyrosine phosphatase is needed for RDGN function, we compared the pattern of the differentiating photoreceptor neurons in the third instar eye fields of *eya*^*+*^*GR* versus *eya*^*E728Q*^*GR* rescued larvae (Fig 2). In agreement with the phenotypes observed in adult animals (Fig 1), qualitative assessment of the field of Elav+ cells in discs from larvae carrying one copy of *eya*^*E728Q*^ in a background of reduced *so*, *dac* or *so* and *dac* dosage showed that it was under-developed compared to wild type (compare Fig 2D, 2F, 2H to 2C, 2E and 2G). Ommatidial organization was also irregular, consistent with the adult rough eye phenotype. Eya protein levels and expression pattern appeared qualitatively similar across the genotypes (Fig 2), as previously reported [27]. We conclude that when other RD proteins are limiting, one copy of Eya^E728Q^ lacks sufficient activity to support normal patterning within the developing eye field.

In the course of these experiments, we were surprised to find that even in animals carrying the normal dose of *so* and *dac*, *eya*^*E728Q*^*GR* was insufficient for normal adult eye formation. Thus in contrast to the published study [27], in our hands, animals carrying one copy of the *eya*^*E728Q*^*GR* transgene but null for endogenous *eya* developed eyes that were not completely wild type (Figs 1A–1D and 2B). We observed a qualitatively similar mild and fully penetrant phenotype in *eya*^*cli*^ homozygotes and in *eya*^*cli*^ */Df(eya)* trans-heterozygotes (Fig 1C), indicating the defects were not caused by homozygosed secondary mutations on the *eya*^*cli*^ chromosome. Consistent with the prior report [27], the eyes of animals heterozygous for *eya*^*D493N*^*GR* were indistinguishable from wild type (Fig 1E and 1F).

Given the different results obtained with the two phosphatase dead GR transgenes, we worried the *eya*^*E728Q*^*GR* chromosome might have accumulated a secondary mutation. We therefore outcrossed to *w*^*1118*^ flies for ten generations and then repeated the analysis. The results matched our initial observations (Fig 1C,1I,1O and 1U). Finally, we sequenced all protein coding exons from the two phosphatase dead BAC transgenes. This analysis confirmed the presence of the expected E728Q and D493N missense changes and the absence of other mutations that would alter the protein sequence. However it remains formally possible that a secondary lesion elsewhere in the *eya*^*E728Q*^*GR* BAC or in a locus closely linked to the transgene insertion site is responsible for the phenotypes observed in our rescue experiments.

While the molecular explanation for the activity differences between *eya*^*E728Q*^*GR* and *eya*^*D493N*^*GR* remains unclear, analogous differences have been noted in other contexts. For example, in *in vitro* phosphatase assays using recombinant murine Eya3, although both the D493N and E728Q equivalent mutations significantly reduced catalytic activity, the former still had measurable phosphatase activity while the latter did not [23]. Greater loss of functionality in *eya*^*E728Q*^ than *eya*^*D493N*^ has also been reported in overexpression-based ectopic eye induction and genetic rescue assays [23,24]. Assuming the two mutations reduce phosphatase activity to different extents in vivo as they do in vitro, then the phenotypic distinctions between *eya*^*E728Q*^*GR* and *eya*^*D493N*^*GR* may reflect a threshold of sensitivity in the requirement for phosphatase activity. Alternatively, the different “phosphatase-dead” missense mutations might disrupt different protein-protein interactions or subtly perturb Eya stability, subcellular localization or other functions in ways our analyses were unable to detect. For example, variations in Eya-So transcriptional functions and interactions could explain the activity differences we noted between *eya*^*E728Q*^*GR* and *eya*^*D493N*^*GR*. Considering the overexpression studies in cultured cells and in the fly that show that “phosphatase-dead” missense mutations do not abrogate So-binding or broadly alter Eya-So transcriptional output [20,23], we favor the hypothesis that the “phosphatase-dead” mutants actually impair catalytic activity in fly cells as they do in vitro and in mammalian cells, and that in genetically sensitized backgrounds, i.e. those in which Eya, So and Dac levels are half normal, this perturbs development. However until a substrate is identified, this caveat must be kept in mind.

### Eya’s tyrosine phosphatase may contribute to the RDGN’s role in survival but is dispensable for fertility

*eya*, *so* and *dac* are considered essential genes, though *dac* null flies occasionally reach adulthood [6,36,38–41], raising the possibility that Eya’s tyrosine phosphatase is also required for survival. To evaluate this, we crossed *eya*^*cli*^*/CyO; eyaGR* to *eya*^*cli*^*/CyO* and then asked how closely the ratio of *eya*^*cli*^*; eyaGR/+* to *eya*^*cli*^*/CyO; eyaGR/+* adult progeny matched the 1:2 Mendelian ratio expected under the null hypothesis that the *eyaGR* transgene fully rescues the lethality associated with complete *eya* loss.

**Fig 3:**
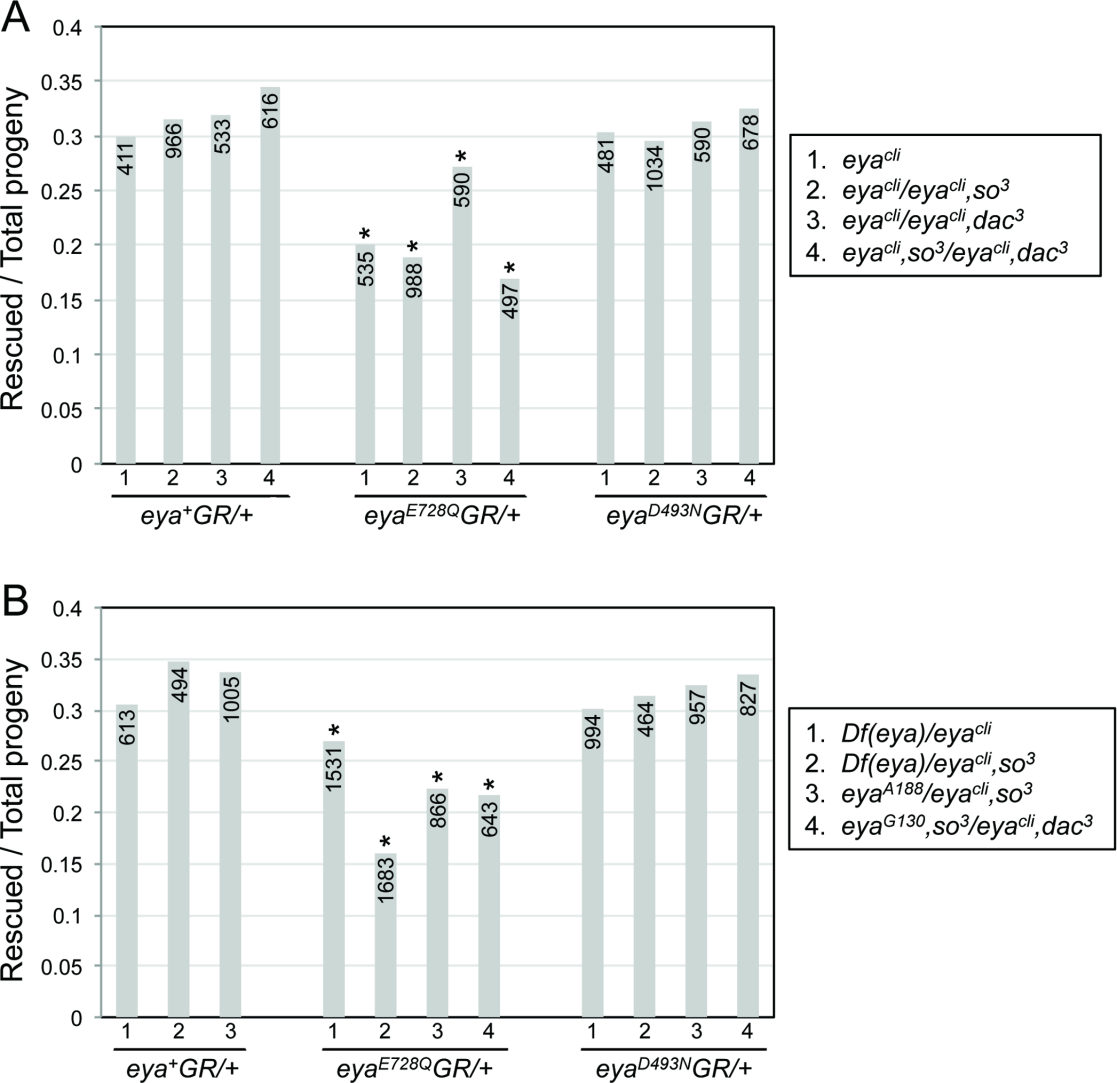
Impaired survival of animals carrying the E728Q “phosphatase-dead” Eya mutation. Bars plot the number of rescued *eya* null progeny divided by the number of total progeny (indicated in each bar) from the cross (see Methods for details). 2^nd^ chromosome genotypes are indicated in key to the right; 3^rd^ chromosome *GR* transgene is indicated below x-axis. *P* values were calculated with a *Χ*^*2*^ test using one degree of freedom; * indicates P < 0.05. (A) One copy of *eya*^*+*^*GR* or *eya*^*D493N*^*GR* fully rescued *eya*^*cli*^ with the predicted Mendelian inheritance ratio of 0.33. In contrast, one copy of *eya*^*E728Q*^*GR* decreased the proportion of rescued progeny below Mendelian expectations. (B) Analogous results were obtained in different *eya* null transheterozygous backgrounds.

*eya*^*E728Q*^*GR* reduced the proportion of homozygous *eya*^*cli*^ animals that eclosed from the expected 0.3 ratio to 0.2 (Fig 3A), suggesting that Eya’s tyrosine phosphatase contributes to biological processes important for viability. *so* or *so*,*dac* heterozygosity exacerbated this phenotype, whereas *dac* heterozygosity partially suppressed. In contrast, animals carrying one copy of *eya*^*D493N*^*GR* eclosed at the expected rate, even in the sensitized backgrounds. We confirmed that these results were independent of second chromosome background by repeating several of the experiments with *eya* null transheterozygotes or null alleles over a deficiency that covers the *eya* locus (Fig 3B). These data indicate that Eya’s tyrosine phosphatase may cooperate with other RD proteins in contexts outside the eye and that “phosphatase-dead” mutants can compromise viability when these proteins are limiting.

**Fig 4:**
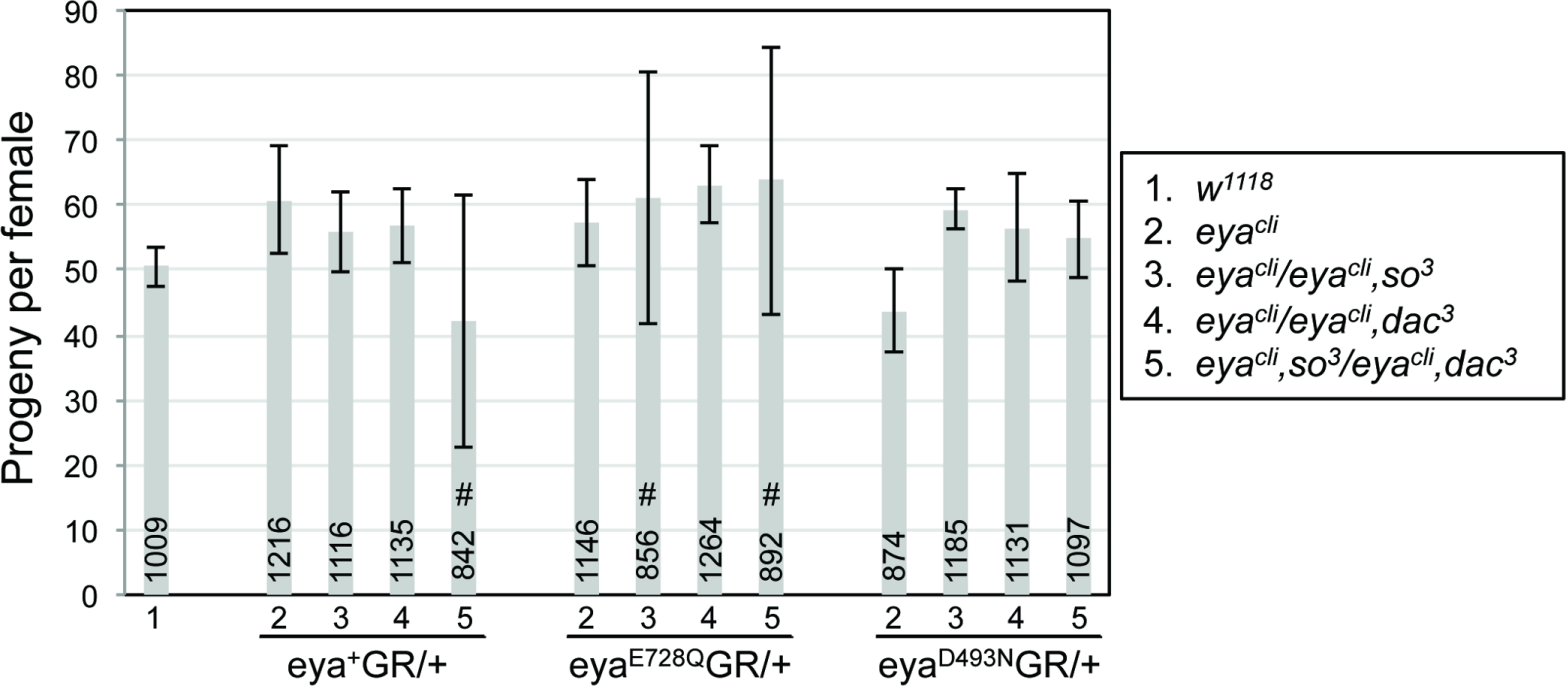
Eya’s tyrosine phosphatase is dispensable for female fertility. For each genotype, 10 pairs of *eya*^*cli*^*; eyaGR/+* females were outcrossed to *w*^*1118*^ males and the total number of adult progeny counted (indicated in each bar; see Methods for details). Data are plotted showing standard error. 2^nd^ chromosome genotypes are indicated in key to the right; 3^rd^ chromosome *GR* transgene is indicated below x-axis. No obvious patterns of differences in fertility were noted. # indicates genotypes for which 3 of the 10 crosses were; although the sterility increased the standard error, for the fertile crosses of those genotypes, progeny numbers were consistent with those of the other genotypes.

We also assessed the role of Eya’s phosphatase in female fertility by counting the number of progeny produced by pairs of *eya*^*cli*^*; eyaGR/+* females outcrossed to wild type males. In contrast to our measurements of retinal development and viability, and in agreement with the earlier study [27], females rescued with the phosphatase dead BAC transgenes were as fertile as controls, including when *so* and/or *dac* levels were reduced (Fig 4). *eya*^*cli*^*; eyaGR/+* males were also fertile in all genetic backgrounds, although we did not quantify or compare fertility rates.

### Concluding remarks

Our work raises the possibility that Eya’s tyrosine phosphatase activity contributes to the robustness of RDGN output during normal retinal development, analogous to its requirement for efficient ectopic eye induction. If correct, this implies that regulation of retinal specification in the eye disc carries sufficient redundancy that it is insensitive to modest genetic stress, including two-fold changes in RDGN protein levels and loss of Eya phosphatase function; only if multiple such perturbations are introduced simultaneously is this buffering capacity overwhelmed. In contrast, we propose that the requirement for higher than usual RDGN output to drive ectopic eye induction makes the system inherently less robust. Thus, while the overexpression-based ectopic eye induction assay may not provide an accurate estimate of RDGN regulatory redundancy during normal retinal development, its inherent lack of robustness makes it exquisitely sensitive, facilitating the discovery of relevant biological regulation. We therefore suggest that the debate as to whether or not *Drosophila* Eya participates in the RDGN as a protein tyrosine phosphatase should remain open and hope our study will motivate further investigations, including a search for physiological substrates.
